# Oral Immunization against PEDV with Recombinant *Lactobacillus casei* Expressing Dendritic Cell-Targeting Peptide Fusing COE Protein of PEDV in Piglets

**DOI:** 10.3390/v10030106

**Published:** 2018-03-01

**Authors:** Xingyu Hou, Xinpeng Jiang, Yanping Jiang, Lijie Tang, Yigang Xu, Xinyuan Qiao, Min Liu, Wen Cui, Guangpeng Ma, Yijing Li

**Affiliations:** 1 Department of Preventive Veterinary Medicine, College of Veterinary Medicine, Northeast Agricultural University, Harbin 150030, China; houxingyu9305@163.com (X.H.); jiangxinpeng03@hotmail.com (X.J.); jiangyanping2017@126.com (Y.J.); tanglijie@neau.edu.cn (L.T.); yigangxu_china@sohu.com (Y.X.); qiaoxinyuan@126.com (X.Q.); cuiwen_200@163.com (W.C.); 2 College of Animal Science and Technology, Northeast Agricultural University, Harbin 150030, China; liumin_707@163.com; 3 Agricultural High Technology Department, China Rural Technology Development Center, Beijing 100000, China

**Keywords:** porcine epidemic diarrhea virus, dendritic cell-targeting peptide, *Lactobacillus casei* vaccine, T-helper cell, mucosal immunity

## Abstract

Porcine epidemic diarrhea (PED) is a highly contagious disease in newborn piglets. In our previous study, a genetically engineered *Lactobacillus casei* oral vaccine (*pPG-COE-DCpep/L393*) expressing a dendritic cell (DC)-targeting peptide fused with porcine epidemic diarrhea virus (PEDV) COE antigen was developed. This vaccine induced significant levels of anti-PEDV specific IgG and IgA antibody responses in mice, indicating a potential strategy against PEDV infection. In this study, *pPG-COE-DCpep/L393* was used for oral vaccination of newborn piglets against PEDV. We then assessed the immune responses and protection efficacy of *pPG-COE-DCpep/L393*. An indirect enzyme-linked immunosorbent assay (ELISA) showed that the recombinant *Lactobacillus* vaccine elicits a specific systemic and mucosal immune response. The T-helper cells mediated by *pPG-COE-DCpep/L393* and PEDV infection display a Th1 phenotype. The histopathological results showed that *pPG-COE-DCpep/L393* promotes lymphocyte proliferation and effectively protects piglets against PEDV infection. The transforming growth factor-β level indicated that the recombinant *Lactobacillus* vaccine plays a role in anti-inflammatory responses in mesenteric lymph nodes during PEDV infection. These results show that *pPG-COE-DCpep/L393* is a potential vaccine against PEDV infection.

## 1. Introduction

Porcine epidemic diarrhea (PED) is a highly contagious disease in piglets. It mainly infects the epithelial cells of the small intestine, and piglets die within seven days. Without an effective immune response against the virus, mortality rate ranges from 70% to 100% in piglets during the first three days of birth [1]. Porcine epidemic diarrhea virus (PEDV) belongs to the *Coronaviridae* family, and is a single-stranded ribonucleic acid (RNA) virus that encodes four structural proteins. The S glycoprotein of PEDV can induce the production of a neutralizing antibody against PEDV infection [2]. The neutralizing epitope region of the PEDV S protein, the collagenase-digested fragment (namely, the CO-26K equivalent, COE), was expressed in transgenic tobacco, and used for immunizing mice, thereby inducing an effective neutralizing antibody response [3]. We therefore hypothesized that a recombinant vaccine using the COE gene would be effective against PEDV infection.

The inactivated and attenuated vaccines (intramuscular route or subcutaneous injection) induced production of maternal antibodies in low titers for a short duration. Most of the maternal antibodies in the milk were digested by gastric acid and pepsin before entering the intestinal tract [4]. Additionally, they did not induce sufficient production of virus-specific IgA antibodies, resulting in inadequate mucosal immunity [5]. Vaccines must effectively provide mucosal protection in the intestinal tract. Mucosal immunity against viral entry into the intestinal epithelial cells was more effective than systemic immunity [6]. Anti-PEDV immunization with an oral vaccine in pregnant swine was better than injected immunization in reducing piglet mortality, and IgA was more important than IgG for protection in the colostrum [7]. Research has suggested that the first line of defense by IgA in the intestine would be more effective than that by IgG for protecting piglets against PEDV infection. Furthermore, dendritic cells (DCs), professional antigen-presenting cells, link humoral and cellular immune responses for homeostasis of the intestinal immune environment, and studies have focused on DC targeting for enhancing antibody titer. DCs can be found in all the lymphoid organs associated with the intestine, such as Peyer’s patches (PPs), isolated lymphoid follicles (ILFs), and mesenteric lymph nodes (MLNs), as well as scattered throughout the subepithelial lamina propria (LP) of both the small intestine and colon [8,9,10].

Lactic acid bacteria (LAB) are considered to be safe microorganisms, with beneficial effects on the health of humans and animals [11]. LAB vaccines can therefore be used as a delivery system to regulate the T-helper cell response and stimulate the secretion of specific IgAs for mucosal immunity [12]. However, no research has been carried out on *Lactobacillus* vaccines for PEDV immunization in piglets. The relationship between inflammation and the LAB of anti-inflammation were also not clear during PEDV infection. *Lactobacilli* are closely associated with many animal species, making them prospective candidates for developing oral vaccines.

Our laboratory has studied recombinant *Lactobacillus* vaccines and their role in the prevention of viral-induced diarrhea in piglets for more than a decade. In our previous study, a genetically engineered *Lactobacillus casei* oral vaccine (*pPG-COE-DCpep/L393*), expressing a DC-targeting peptide fused with a PEDV COE antigen, was developed [13]. The results showed that oral vaccination with *pPG-COE-DCpep/L393* can induce significant levels of anti-PEDV systemic IgG and mucosal IgA antibody responses in mice. However, the effectiveness of IgA as the first line of defense, whether IgA-based mucosal immunity would neutralize PEDV, and the clinical protective effect, were not studied in piglets. The aim of this study was to characterize the immunological mechanisms involved when piglets are vaccinated against PEDV infection with the *Lactobacillus* vaccine.

## 2. Materials and Methods

### 2.1. Virus, Bacterium, and Cell Line

*Lactobacillus casei* strain *ATCC 39392 (L393)* and the recombinant *Lactobacillus* (*pPG-COE-DCpep/L393*, *pPG/L393*) were prepared and constructed as described previously [14]. PEDV LJB/15 was previously obtained by our laboratory. African green monkey kidney cells (Vero) were used for culturing PEDV. All Vero cells were cultured in Dulbecco’s-modified Eagle’s medium (DMEM, Gibco, Carlsbad, CA, USA) and supplemented with 10% fetal bovine serum (FBS, Gibco). At the same time, plaque assays were used to quantify the amount of PEDV. Virus stocks were harvested using Vero cells and the virus titer was 10^6^ plaque-forming units (PFU)/mL.

### 2.2. Vaccination Programs

PEDV-seronegative, crossbred, large white piglets were obtained from a local farm after birth, and were housed in specialized stainless-steel cages, which were maintained in negative-pressure isolation rooms and fed commercial sterile milk with fresh water. All the pigs were confirmed to be negative for PEDV, from both pathogenic and serological tests by polymerase chain reaction (PCR) [14] and ELISA [13], respectively. All animal experiments were approved by the Experimental Animal Care Ethics Committee of Northeast Agricultural University (2017NEFU-219, 13 April 2017).

The piglets were randomly assigned to five groups (Groups A–E, all *n* = 5). The piglets received *pPG-COE-DCpep/L393* or phosphate-buffered saline (PBS) alone in a different vaccination programs via an oral route, with 10^10^/mL colony-forming units (CFU) (for *pPG-COE-DCpep/L393*) in PBS. The immunization program was based on previous research on dosage and time points for immunization with recombinant *Lactobacillus* in piglets [15]; Group A (1 mL *pPG-COE-DCpep/L393*/Day 0); Group B (2 mL *pPG-COE-DCpep/L393*/Day 0); Group C (2 mL *pPG-COE-DCpep/L393*/Day 2); Group D (4 mL *pPG-COE-DCpep/L393*/Day 2); Group E (1 mL PBS). Groups A and B received an oral vaccine with different dosages of *pPG-COE-DCpep/L393* immediately after birth. Groups C and D received an oral vaccine with different dosages of *pPG-COE-DCpep/L393* on the second day after birth. Serum samples were collected from each group on days 3, 5, 7, 9, and 11 after vaccination for testing for specific IgG. Mucus extracts were collected using rectal swabs from each group on days 3, 5, 7, 9, and 11 after vaccination, to test for specific IgA. All samples were stored at −80 °C for subsequent analysis. The most effective vaccination program was used to test protective efficacy in the last vaccination. Newborn piglets were randomly allocated to three groups, and received 2 mL *pPG-COE-DCpep/L393* (*n* = 10), *pPG/L393* (*n* = 10), and PBS (*n* = 15). Serum samples were collected from each group on day 5 after vaccination for testing for specific IgG. Mucus extracts were collected using rectal swabs from each group on day 5 after vaccination, to test for specific IgA. The experiment was repeated three times.

### 2.3. Enzyme-Linked Immunosorbent Assay

Indirect ELISAs were performed by coating alternate wells with 250 ng/well of COE protein for 12 h at 4 °C. Each well was blocked with 200 μL of skim milk (PBS plus 5% skim milk), and incubated overnight at 4 °C. The plates were washed three times with 200 μL of PBST (PBS plus 0.1% Tween20). Mucus extracts and serum samples were diluted in triplicate and incubated for 1.5 h at 37 °C. Serum was diluted from 1:50 to 1:800, and mucus extracts were diluted from 1:100 to 1:3200. The plates were washed three times and horseradish peroxidase-conjugated (HRP) goat anti-piglet IgG or IgA antibody (Abcam, Cambridge, UK) was added, then incubated for 1.5 h in the dark, and then washed three times. The substrate *o*-phenylenediamine dihydrochloride (OPD) was used as the chromogen. The reaction was stopped using 2 N H_2_SO_4_ and quantified spectrophotometrically at 490 nm with an ELx800 microplate reader (BioTek, Winooski, VT, USA). The performance of each group of plates was standardized using a panel of reference IgA/IgG negatives and positives. P/N ratios > 2 were considered antibody-positive.

### 2.4. Th Cell Analysis before and after Infection

Flow cytometry (FCM) was used to analyze the frequencies of peripheral blood T lymphocytes (10^6^ cells mL^−1^) as previously described [16]. T cells were harvested and washed three times with PBS (pH 7.2). In the presence of monensin (2 μM), single-cell suspensions were stimulated using ionomycin (2 μg mL^−1^) for 4 h. The cells were surface-labelled with cluster of differentiation 4 (CD4) (fluorescein isothiocyanate (FITC)-conjugated) antibodies, fixed, permeabilized, and intracellularly labelled. The cells were stained with anti-interferon (IFN)-γ (P-phycoerythrin (PE)-conjugated) from Th1 cells, and anti-interleukin (IL)-4 (PerCP-Cy5.5-conjugated) (Abcam) from Th2 cells. Cells were then washed three times with PBS (pH 7.2), and 200 μL cell suspensions (0.01 M PBS) was analyzed on a fluorescence-activated cell sorting (FACS) Calibur cytometer. Cell numbers as assessed using flow cytometry were higher than 10^4^ in every sample.

To analyze the immune cell frequencies in peripheral blood T lymphocytes, flow cytometry analysis of the percentage of T helper cell phenotype, as gating CD4+-IFN-γ+ and CD4+ IL-4+, was performed. All animals in the respective groups were analyzed, and the experiment was repeated three times. All piglets were tested by FCM.

### 2.5. Protective Efficacy

Three groups of piglets (*pPG-COE-DCpep/L393*, *pPG/L393*, and PBS) were chosen to be infected with PEDV, to analyze the protective efficacy of the vaccination. The *pPG-COE-DCpep/L393* (*n* = 10), *pPG/L393* (*n* = 10), and PBS (*n* = 10) groups were infected with PEDV using oral-gastric gavage, with a 5 mL dose of PEDV LJB/15 strain (1 × 10^6^ PFU mL^−1^). The other five piglets in the control group were used as negative controls. The health status and weight change of the piglets were monitored. All piglets were euthanized, and dissection was performed on the fourth day post-infection (DPI). The protective efficacy data were then analyzed.

### 2.6. Gross Lesion and Histopathological Examinations

Intestinal tissues were collected from the standardized areas of the jejunum within 10 to 15 min post-mortem. The intestinal tissue samples were fixed and stained according to routine histological methods. After immediate fixation in 10% buffered formalin, the samples were embedded in paraffin wax. Paraffin sections (6 µm) were stained with hematoxylin–eosin (H&E) for routine histological examination. The samples were all histopathological examined using light microscopy (Olympus, Tokyo, Japan).

### 2.7. Immunofluorescence Assay (IFA)

The tissues of the small intestine were frozen at −80 °C, sectioned with a cryostat, and fixed on a glass slide for 10 min in acetone. The slides were fixed, permeated for 10 min at room temperature, washed three times with PBS, and blocked with 0.3% bovine serum albumin (BSA) in PBS at 37 °C for 1 h. Samples were then incubated separately with rabbit polyclonal antibody directed against PEDV (1:100) for 30 min at room temperature, and treated with goat anti-rabbit IgG H&L antibody (FITC) (Abcam) for 30 min at 37 °C. Finally, the samples were washed and examined under a fluorescence microscope (Leica, Wetzlar, Germany).

### 2.8. Real-Time RT-PCR (qRT-PCR) Analysis

Real-time PCR was performed in triplicate to determine the expression of the cytokines in the spleen and intestinal tissues, using the ABI Prism 7500 sequence detection system (Applied Biosystems, Foster City, CA, USA) with SYBR green fluorescence detection. Total RNA samples were extracted from splenic and mesenteric lymph nodes using TRIzol (Gibco) reagent according to the manufacturer’s instructions. Total RNA was reverse transcribed into complementary DNA (cDNA) using Moloney murine leukemia virus (M-MLV), reverse transcriptase, and oligo(dT)_18_ primers (Takara, Dalian, China). The cDNA was prepared for real-time qRT-PCR using a SYBR^®^ qPCR Mix Reagent Kit (Takara). Absolute quantification real-time PCR was performed to determine the copy number of PEDV in feces. PEDV shedding was detected in the *pPG-COE-DCpep/L393*, *pPG/L393*, and PBS groups at every 12 h timepoint. Feces were diluted 1:10 (*w*/*v*) in minimum essential media (MEM). Fecal suspensions were clarified by centrifugation for 10 min at 4000× *g* to eliminate fecal debris. The RNA was extracted from 200 μL of sample and reverse transcription performed as described above. A standard curve was generated by plotting the threshold values against the serially diluted plasmid DNA encoding the PEDV M protein fragment. Real-time quantitative PCR was utilized to quantify and compare the products of interest (toll-like receptor (TLR)-4, TLR-9, TGF-β, and TNF-α) between the vaccination groups and the PBS group after PEDV infection. β-actin was used as the internal control. The relative expression levels of the cytokines were compared with those of β-actin and uninfected piglets, using the 2^−ΔΔCt^ method. Specific primers for TLR-4, -9, TGF-β, TNF-α, β-actin, and PEDV-M sequences are shown in Table 1 [17,18,19]. All piglets were tested and for each piglet, three samples were tested.

### 2.9. Statistical Analysis

A paired *t*-test was used to determine significant differences in the intestinal mucus and serum antibody titers of different groups. Cytokine expression and Th cell analysis were compared using a one-way analysis of variance (ANOVA) general linear model followed by Duncan’s multiple range test. SPSS 16.0 (SPSS, Chicago, IL, USA) was used to perform all the analyses, and *p*-values < 0.05 were considered statistically significant. *p*-values are indicated as follows: * *p* < 0.05 and ** *p* < 0.01. All results in the figures are presented as the mean ± the standard error of the mean (SEM).

## 3. Results

### 3.1. Production of Mucosal and Humoral Immunity

**Figure 1:**
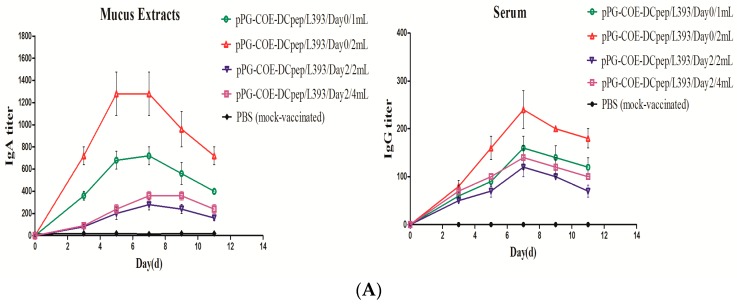

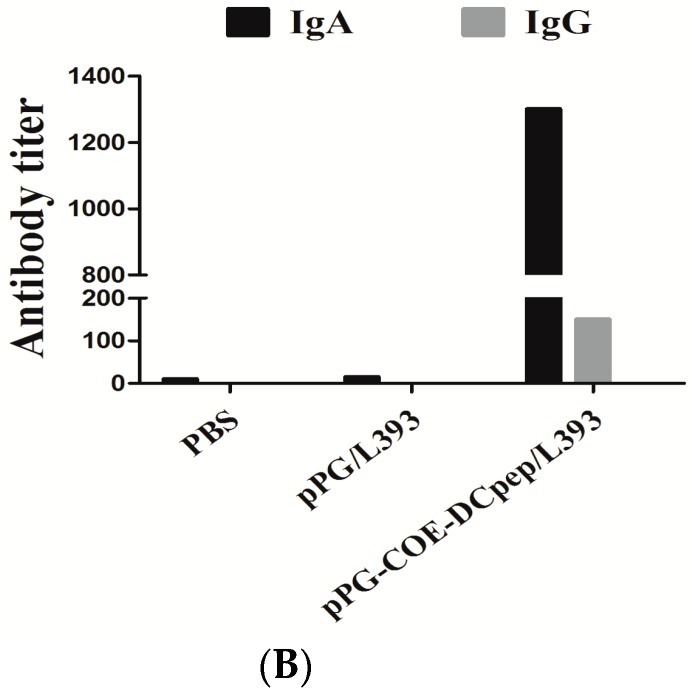
Specific secretory immunoglobulin (Ig) levels. The piglets received *pPG-COE-DCpep/L393* (10^10^ CFU in 1 mL of phosphate-buffered saline (PBS) and PBS alone) for the different vaccination programs, via an oral route; Group A (1 mL *pPG-COE-DCpep/L393*/Day 0); Group B (2 mL *pPG-COE-DCpep/L393*/Day 0); Group C (2 mL *pPG-COE-DCpep/L393*/Day 2); Group D (4 mL *pPG-COE-DCpep/L393*/Day 2); Group E (1 mL PBS). (**A**) Mucus extracts were collected using rectal swabs from the groups administered with *pPG-COE-DCpep/L393* on days 3, 5, 7, 9, and 11 post-vaccination. The results represent the IgA titer ± standard errors of the means in each group. Serum was collected from the groups administered with *pPG-COE-DCpep/L393* on days 3, 5, 7, 9, and 11 post-vaccination. The results represent the IgG titer ± standard errors of the means in each group. (**B**) The immune effects of *pPG-COE-DCpep/L393*, *pPG/L393*, and PBS. The piglets received 2 mL *pPG-COE-DCpep/L393*, *pPG/L393* (10^10^ CFU in 1 mL of PBS), and PBS alone, for the different vaccination programs via an oral route. Mucus extracts were collected using rectal swabs from different groups on five days post-vaccination. The results represent the IgA titer ± standard errors of the means in each group. Serum was collected from the groups administered *pPG-COE-DCpep/L393* five days post-vaccination.

To measure the IgA titer, mucus extracts were collected to study the specific antibodies of IgA (Figure 1A). The specific IgA peaked at five days post-vaccination (1:1400) in Group B, which was significantly higher than that in the other groups. These results suggested that the piglets in Group B had the highest IgA levels. Meanwhile, we measured the levels of IgG in the serum (Figure 1A). The piglets in Group B also had the highest specific IgG levels (1:250). This showed that *pPG-COE-DCpep/L393* could elicit specific systemic immunity. The specific IgG titer was the highest at seven days post-vaccination, which was significantly higher than that in the other groups. No significant specific IgA or IgG titers were observed in the PBS group. These results indicated that oral *pPG-COE-DCpep/L393* effectively induced systemic and mucosal immunity in piglets. Group B, immunized with 2 mL of *pPG-COE-DCpep/L393*, showed the strongest immune effectivity in the newborn piglets, which was also the best immunizing program.

This program was used for re-immunization and for analyzing the protective efficacy. The results showed that the IgA titer of *pPG-COE-DCpep/L393* in the mucus extracts was significantly higher than that of *pPG/L393* or PBS. The IgG titer of *pPG-COE-DCpep/L393* in the serum was also significantly higher than that of *pPG/L393* and PBS. *pPG-COE-DCpep/L393* therefore inducing specific systemic and mucosal immunity in piglets.

### 3.2. The Percentage of T-Helper Cells by pPG-COE-DCpep/L393

**Figure 2:**
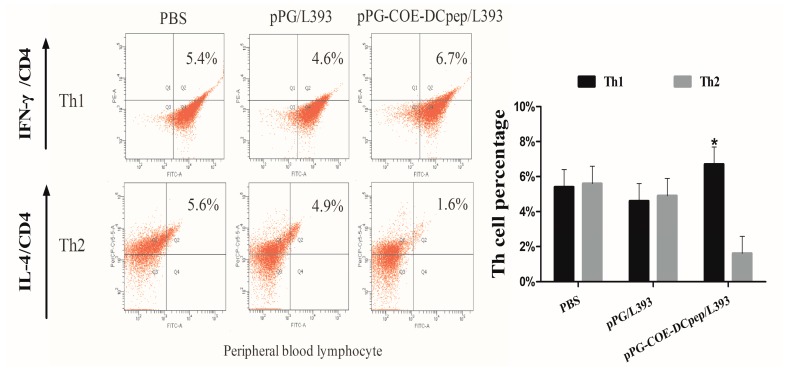
The percentage of T-helper cells induced by *pPG-COE-DCpep/L393*. Blood lymphocytes from piglets immunized with 2 mL of *pPG-COE-DCpep/L393*, *pPG/L393*, and PBS, were analyzed by flow cytometry (FCM) assay to evaluate the percentages of Th1 and Th2. Flow cytometry analysis was carried out on CD4+ cells labeled with fluorescein isothiocyanate (FITC), anti-interferon (IFN)-γ conjugated with P-phycoerythrin (PE), and anti-interleukin (IL)-4 labeled with PerCP-Cy5.5. The data were acquired using a fluorescence-activated cell sorting (FACS) Calibur cytometer. To analyze the immune cell frequencies in peripheral blood T lymphocytes, flow cytometry analysis of the percentage of T helper cell phenotype, as gating cluster of differentiation 4 (CD4+ IFN-γ+ and CD4+ IL-4+, was performed. The results are represented as the mean ± SEM of three independent experiments. Asterisks indicate a significant difference between the percentage of T-helper subtypes of the different groups (* *p* < 0.05).

To determine the effects of *pPG-COE-DCpep/L393* on T-helper cells after vaccination, we analyzed the conversion of T-helper cells using a FACS Calibur cytometer. The result showed the percentage of Th1 was significantly higher than that of Th2 in the *pPG-COE-DCpep/L393* group (*p* < 0.05) (Figure 2). The *pPG/L393* and PBS groups did not show a significant change. The predominant phenotype of T cells mediated by *pPG-COE-DCpep/L393* therefore displays a Th1 phenotype.

### 3.3. Protective Efficacy

**Figure 3:**
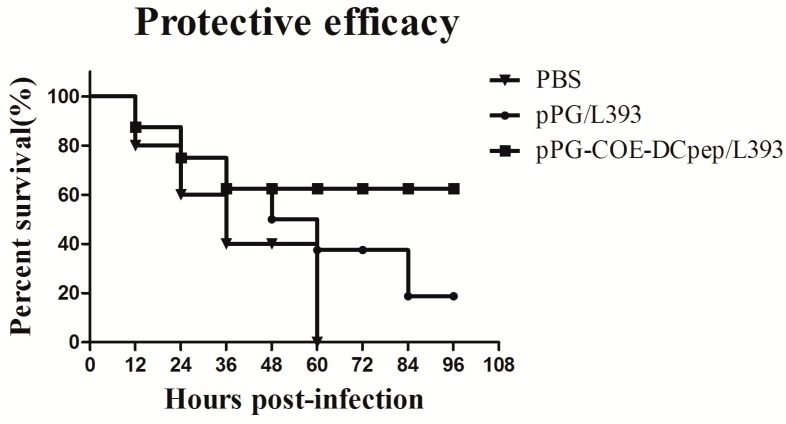
Protection after Porcine epidemic diarrhea virus (PEDV) infection. The *pPG-COE-DCpep/L393*, *pPG/L393*, and PBS groups were orally infected with PEDV (1 × 10^6^ PFU/mL) on the fifth day post-vaccination. All piglets of the *pPG-COE-DCpep/L393*, *pPG/L393*, and PBS groups were euthanized, and dissection at four days post-infection (DPI).

After vaccination, the PEDV LJB/15 strain was used to infect the *pPG-COE-DCpep/L393*, *pPG/L393*, and PBS groups. Sixty percent of the piglets were protected in the *pPG-COE-DCpep/L393* group (Figure 3). The protective efficacy of *pPG/L393* and PBS was lower than that of *pPG-COE-DCpep/L393*. All piglets in the PBS group died within 60 h. The piglets in the *pPG-COE-DCpep/L393* group showed lower weight loss than those in the *pPG/L393* and PBS groups (Table 2). All piglets had diarrhea with yellow feces coating the skin and hair. In the dead piglets, the intestinal lumens were empty, filled with large amounts of yellowish foamy fluid, and the walls of the small intestines had thinned.

### 3.4. Gross Lesion and Histopathological Examinations

**Figure 4:**
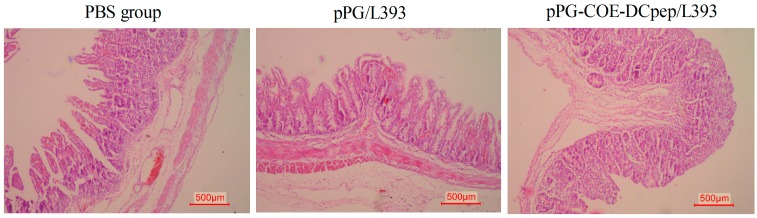
*pPG-COE-DCpep/L393* protects organs from porcine epidemic diarrhea virus (PEDV)-induced destruction. Histopathological examinations of the H&E-stained small intestinal tissues were analyzed after euthanizing the piglets for dissected examination at four DPI. PEDV-infected pigs received a 5-mL dose of 1 × 10^6^ PFU/mL via oral-gastric gavage. The figure shows the tissue of the jejunum sections from the *pPG-COE-DCpep/L393*, *pPG/L393*, and PBS groups. The histological pictures are representative of sections derived from each group.

The small intestine tissues from the different groups were used for pathological examination (Figure 4). Histological samples from the PBS groups showed small intestines that were characterized by marked degeneration, necrotic enterocytes, hyperemia of intestinal villi, and vacuolization, compared with the *pPG-COE-DCpep/L393* group. The intestinal villi of the jejunum in the PBS group were shorter than in the *pPG-COE-DCpep/L393* group. Lymphocyte cell proliferation was observed in the *pPG-COE-DCpep/L393* group in the jejunum, in which pathological changes were not observed. Compared to that of the PBS group, superficial villous enterocyte swelling was not severe in the *pPG-COE-DCpep/L393* group. Severe inflammatory responses were found after PEDV infection in the *pPG/L393* and PBS groups. The *pPG-COE-DCpep/L393* group showed the lowest inflammatory responses, compared with the PBS and *pPG/L393* groups.

### 3.5. Application of Real-Time RT-PCR to Clinical Samples

**Figure 5:**
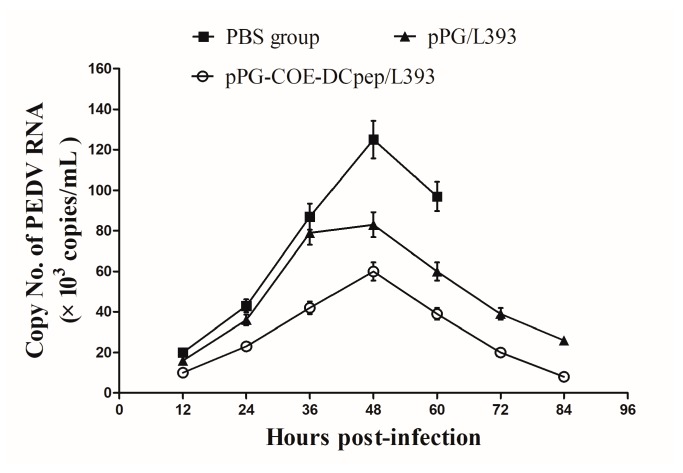
Real-time reverse transcriptase polymerase chain reaction (RT-PCR) of clinical samples. PEDV shedding in the *pPG-COE-DCpep/L393*, *pPG/L393*, and PBS groups, was collected every 12 h. Fecal samples were taken until death (1–4 DPI). Total RNA was extracted from the feces. The PEDV copy number was determined by real-time qPCR analysis. The results are represented as the mean ± standard error of mean (SEM) of three independent experiments.

Fecal samples were collected from all piglets to assess the copy number of the PEDV RNA in feces. The load of PEDV fecal shedding was 2.1 × 10^4^ copies at 12 h in the PBS group. The highest copy number in the PBS group was detected at 48 h, peaking at 1.25 × 10^5^ copies, which gradually decreased until piglet death (Figure 5). The *pPG-COE-DCpep/L393* groups had lower copy numbers than the PBS and *pPG/L393* groups. The RNA copy numbers reached their highest level (5.5 × 10^4^ copies) at 48 h in the *pPG-COE-DCpep/L393* group.

### 3.6. Immunofluorescence Assay (IFA)

**Figure 6:**
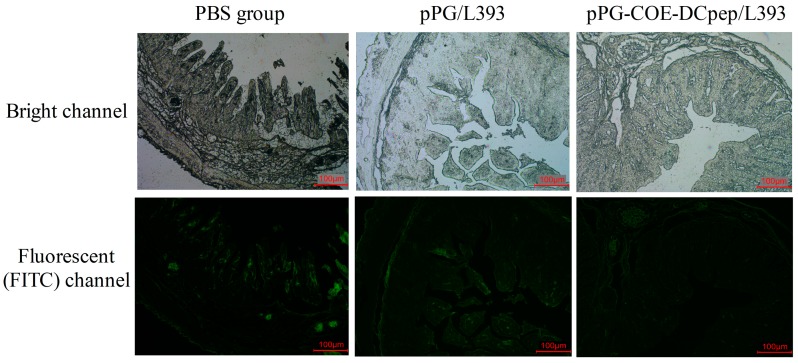
PEDV distribution in the jejunum. The piglets were orally infection with PEDV (1 × 10^6^ PFU/mL) via oral-gastric gavage. The PEDV distribution patterns were detected in the villous by an immunofluorescence assay using rabbit polyclonal antibody against PEDV. The top pictures are from the light channel, and the bottom pictures are from the fluorescent (FITC) channel. The upper and lower panel were from the same section. The pictures are representative of sections derived from each group.

We used an immunofluorescence assay (IFA) to verify the viral antigen distribution patterns in the villous, using a rabbit polyclonal antibody against PEDV (Figure 6). The viral antigens were mainly detected in villous epithelial cells. We found that the viral antigens of the PBS group in the jejunum were more extensive than in the *pPG-COE-DCpep/L393* and *pPG/L393* groups. The weakest PEDV antigen signals were detected in the *pPG-COE-DCpep/L393* group, compared with the *pPG/L393* group infected with PEDV. The result indicates that the severity of PEDV infection in the *pPG-COE-DCpep/L393* group was not as high as that in the PBS and *pPG/L393* groups.

### 3.7. The Percentage of T-Helper Cells after PEDV Infection

**Figure 7:**
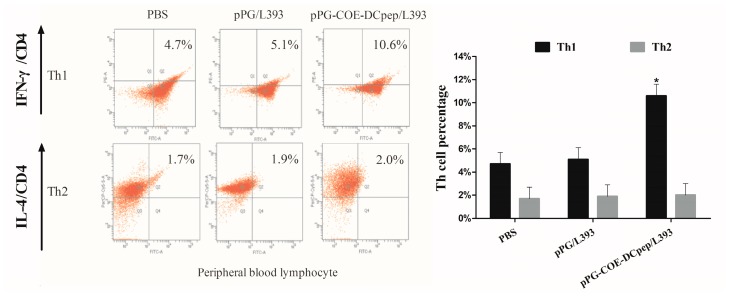
The percentage of T-helper cells after PEDV infection. The piglet groups immunized with *pPG-COE-DCpep/L393*, *pPG/L393*, and PBS were infected with PEDV (1 × 10^6^ PFU/mL) via oral-gastric gavage. T lymph cells of the piglets were collected from peripheral blood, and subjected to an FCM assay, to evaluate the Th1 and Th2 percentages. Flow cytometry analysis of CD4+ cells labeled with FITC, anti-interferon (IFN)-γ conjugated with PE, and anti-interleukin (IL)-4 labeled with PerCP-Cy5.5. To analyze the immune cell frequencies in peripheral blood T lymphocytes, flow cytometry analysis of the percentage of T helper cell phenotype, as gating CD4+ IFN-γ+ and CD4+ IL-4+ was performed. Statistical differences (* *p* < 0.05) are indicated by an asterisk. The results are represented as the mean ± SEM of three independent experiments.

T-helper cells were counted to determine the type of immune responses modulated by oral vaccination after PEDV infection. As shown in Figure 7, the percentage of Th1 cells induced by *pPG-COE-DCpep*/*L393*, which had a significantly higher compared with Th2 cells. Compared with the percentage of Th1 and Th2, the percentage of Th1 cells in all groups was significantly higher than Th2 cells (*p* < 0.05). The predominant phenotype of T cells mediated by PEDV infection is therefore a Th1 phenotype.

### 3.8. Cytokine Expression

**Figure 8:**
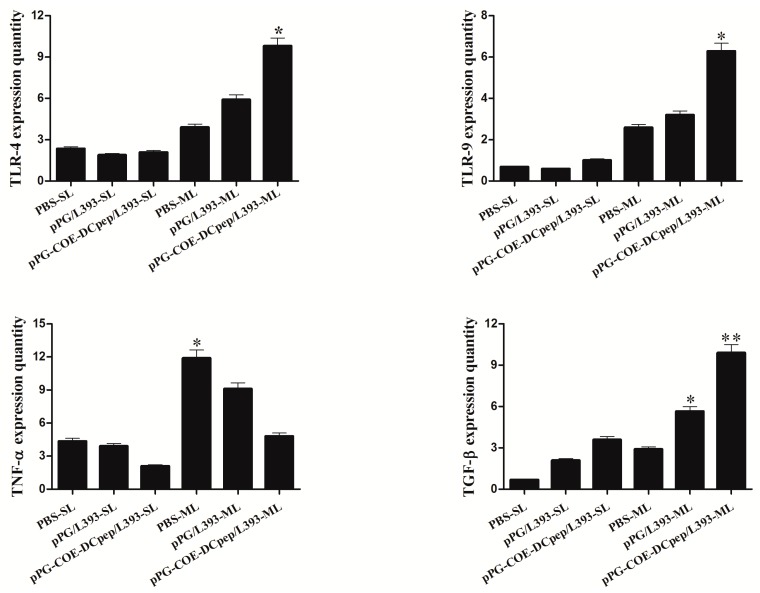
Cytokines and toll-like receptor (TLR) expression. RNA expression was analyzed in splenic lymphocytes (SL) and mesenteric lymph node (ML) of the *pPG-COE-DCpep/L393*, *pPG/L393*, and PBS groups. Total RNA was used to analyze the expression of TNF-α, TGF-β, TLR-4, and TLR-9. The differences between means were considered as significant at * *p* < 0.05 and very significant at ** *p* < 0.01.

The cytokine mRNA expression in splenic lymphocytes and mesenteric lymph nodes upon infection, is shown in Figure 8. In the mesenteric lymph nodes, significantly higher expression levels of TLR-4, 9 were observed in the *pPG-COE-DCpep/L393* group compared to the *pPG/L393* and PBS groups, but TLR-4, 9 did not show a markedly increased expression in splenic lymphocytes. With respect to the splenic lymphocytes and mesenteric lymph nodes, the expression of TNF-α in the *pPG-COE-DCpep/L393* group was significantly lower than that in the *pPG/L393* and PBS groups, but the expression in the mesenteric lymph nodes of the PBS group was significantly increased compared with that of the *pPG-COE-DCpep/L393* group. Both *pPG-COE-DCpep/L393* and *pPG/L393* groups could effectively reduce inflammation during PEDV infection. Both *pPG-COE-DCpep/L393* and *pPG/L393* groups showed a significant increase in TGF-β expression, compared with the PBS group in the mesenteric lymph nodes (*p* < 0.01).

## 4. Discussion

Our study compared the difference in the immune responses induced by four programs of oral vaccines, and then evaluated the protective immune effects. The results showed that the piglets in Group B had the best systemic and mucosal immune responses. The above results indicated that 2 mL of *pPG-COE-DCpep/L393* stimulated specific mucosal immunity in the newborn piglets. In addition, specific IgG in Group B was induced to the highest levels, making this the optimal immune program. The oral immunization with recombinant *Lactobacillus* stimulated systemic immunity and mucosal immunity, and DC-targeting peptide enhanced the antibody titer of IgG and IgA. The best vaccination program was also used to analyze the type of immunity induced in the *pPG-COE-DCpep/L393* group, protecting the piglets against PEDV infection.

The results indicated that oral *pPG-COE-DCpep/L393* vaccination relieved the disease symptoms and that systemic immunity was stimulated by specific mucosal immunity. The quantitative reverse-transcriptase PCR (qRT-PCR) of the copy number of the PEDV RNA shown that the immunized group has the lowest of number of the copy number of the PEDV, which pointing to the possibility that PEDV was neutralized by specific IgA antibodies. *Lactobacillus* colonizes the digestive tract and other mucosal epithelial cell layers, and the expressed antigen of *pPG-COE-DCpep/L393* was more easily captured by membranous (M) cells and immune cells. *Lactobacillus* could help to induce stronger non-specific mucosal immune responses. The non-specific mucosal immunity can enhance the permeability of intestine to aid the removal of PEDV by macrophages and DCs in the intestine [20]. However, our results proved that *pPG-COE-DCpep/L393* effectively reduces mortality, compared with the PBS and *pPG/L393* groups.

During PEDV infection, the *pPG-COE-DCpep/L393* group showed greatly reduced weight loss compared with the PBS and *pPG/L393* groups. In large-scale swine farms, the piglet’s weight from birth to weaning is a factor that is associated with the future quality of piglets. The protection rate of *pPG-COE-DCpep/L393* did not reach 100%, but it had a protective effect on the weight of the piglets, which is an advantage for farm economics. PEDV infection causes severe atrophic enteritis and villous fusion [21,22]. Histopathological analysis showed severe pathological changes in the villous epithelial cells of the PBS-infected group, compared with that of the *pPG-COE-DCpep/L393* group. Lymphocyte proliferation in the intestinal lamina propria was only observed in the *pPG-COE-DCpep/L393* group.

Kim et al. evaluated the effective clinical results of medicinal herbs during PED infection in the atrophied villus structure and crypt hyperplasia [23]. These results are consistent with the histopathological data from our research, indicating that *pPG-COE-DCpep/L393* has a beneficial effect on the integrity of the intestinal mucosal structure, in piglets infected with PEDV. In addition, the IFA results show that the viral antigen in the immunized group was significantly lower than in the PBS and *pPG/L393* groups. This result indicates that *pPG-COE-DCpep/L393* was effective in controlling PEDV-infecting enterocytes.

T-helper cells were thought to be limited to two major subsets, Th1 and Th2 cells, which was based on their production of specific cytokines (IFN-γ and IL-4, respectively). Th1 cells are crucial for host defense against intracellular pathogens of cellular immunity, whereas Th2 cells are known to be important for the elimination of helminthic parasites with humoral immunity. The Th1/Th2 paradigm is useful for classifying the immune responses that occur in the elimination of microbial pathogens, such as cellular immunity and humoral immunity [24,25]. We analyzed T-helper cell immune responses using peripheral blood T lymphocytes, before and after infection. The DNA plasmids vaccine of PEDV increased the proliferation of CD4+ and CD8+ T lymphocyte subgroups, and increased IFN-γ production in mice [26]. Our previous research showed that recombinant *Lactobacillus* was effective against TGEV infection, via the Th2 immune response. However, there are a number of studies that show that IFN-γ inhibits PEDV infection, and that a close relationship exists between DCs and IFN-γ during PEDV infection [27,28]. We found that the recombinant *Lactobacillus* could weaken the PEDV infection in the form of IgA and IgG, which were up-regulated by the DC-targeting peptide as a molecular adjuvant.

Cytokines have both pro- and anti-inflammatory properties, both of which play critical roles in pathogenesis and infectious diseases [29,30]. Our research first assessed cytokine profiles in piglets that were orally immunized with *pPG-COE-DCpep/L393* after challenging with PEDV. We evaluated the expression of TLR-4, TLR-9, TNF-α, and TGF-β. We tested cytokine expression in the spleen and mesenteric lymph nodes. TLRs are the pattern recognition receptors of the innate immune system, and recognize pathogens through pathogen-associated molecular patterns (PAMPs) [31]. TLRs recognize the pathogen-derived molecules at the earliest phase in the promotion of the immune response [32]. Our results showed that the expression of TLR-4 and TLR-9 in the mesenteric lymph nodes of the *pPG-COE-DCpep/L393* group, was much higher than that in the PBS and *pPG/L39* groups. TLR-4 was activated by bacterial lipopolysaccharide (LPS) and lipoteichoic acid (LTA), while TLR-9 recognizes unmethylated CpG motifs in bacterial DNA [33]. These studies have shown that TLR-9 is induced by CD4+ T cells, which are prolonged by the T cell [34,35]. Carvalho et al. reported that a higher expression of TLR-9 resulted in increased B cell proliferation, in response to CpG DNA [36]. The proliferation of T cells and B cells enhanced the IgA and IgG titers. All of these findings provide evidence that *pPG-COE-DCpep/L393* can function as a bacterial carrier for antigen delivery, enhancing TLR expression and increasing antibody secretion in local immunity.

TNF-α is a proinflammatory cytokine that is elevated in regions of tissue injury and inflammatory diseases. TNF-α links cell proliferation and survival, with different modalities of cell death, which are intricately linked to the epithelial response to injury [37]. TNF-α expression in the splenic lymphocytes and mesenteric lymph nodes of the *pPG-COE-DCpep/L393* group was significantly lower than that in the *pPG/L393* and PBS groups. TGF-β is a ubiquitous modulator of cellular responses, which also regulates numerous responses in monocytes, such as activation, host defense, and chemotaxis [38]. During infection, higher levels of TGF-β may inhibit potential injuries resulting from increased inflammatory responses at sites of infection [39]. In our study, TGF-β levels in the *pPG-COE-DCpep/L393* and *pPG/L393* groups were significantly higher in mesenteric lymph nodes than in the PBS group, and there was no significant difference in their levels in splenic lymphocytes. Anti-inflammatory effects would therefore result in marked protection of intestinal epithelial cells against PEDV infection. These results suggest that *pPG-COE-DCpep/L393* induces anti-inflammatory responses in mesenteric lymph nodes.

To the best of our knowledge, this is the first study to evaluate the immunoprophylactic effects of a recombinant *Lactobacillus casei* expressing DC-targeting peptide fused with a COE protein, against PEDV in piglets. The *pPG-COE-DCpep/L393* vaccine induced specific systemic and mucosal immunity, protecting piglets against PEDV infection. The protective efficacy reached 60%. In addition, we found that the *pPG-COE-DCpep/L393* could also inhibit TNF-α expression, and promote TGF-β expression, thereby inhibiting inflammation in mesenteric lymph nodes.

## Figures and Tables

**Table 1:** Primer sequences used in this study.

Gene	Sequence (5′-3′) Forward	Sequence (5′-3′) Reverse	Accession No. ^a^
TLR4	CTCTGCCTTCACTACAGAGA	CTGAGTCGTCTCCAGAAGAT	AB078418
TLR9	GTGGAACTGTTTTGGCATC	CACAGCACTCTGAGCTTTGT	AB071394
TGF-β	CACGTGGAGCTATACCAGAA	TCCGGTGACATCAAAGGACA	Y00111
TNF-α	ATTCAGGGATGTGTGGCCTG	CCAGATGTCCCAGGTTGCAT	JF831365.1
β-actin	CATCACCATCGGCAACGA	GCGTAGAGGTCCTTCCTGATGT	U07786
PEDV-M	GGTTCTATTCCCGTTGATGAGGT	AACACAAGAGGCCAAAGTATCCAT	AF353511.1

^a^ The sequences of the two primers were checked using NCBI BLAST Software (NCBI, Bethesda, MD, USA), and no significant alignment with any other animal virus gene was found.

**Table 2:** Weight change of piglets.

Group	Mean Weight Gain after Vaccination (kg)	Mean Weight Gain after Infection (kg)
PBS	1.39 ± 0.13	−0.65 ± 0.08
*pPG-COE-DCpep/L393*	1.91 ± 0.27 *	−0.18 ± 0.12 **
*pPG/L393*	1.77 ± 0.14 *	−0.43±0.17 *

* *p*-Value < 0.05 was considered considered statistical difference; ** *p*-value < 0.01 was considered significant statistical difference.
